# Development of a Simulation Model for Fluorescence-Guided Brain Tumor Surgery

**DOI:** 10.3389/fonc.2019.00748

**Published:** 2019-08-16

**Authors:** Daniel Valli, Evgenii Belykh, Xiaochun Zhao, Sirin Gandhi, Claudio Cavallo, Nikolay L. Martirosyan, Peter Nakaji, Michael T. Lawton, Mark C. Preul

**Affiliations:** ^1^Department of Neurosurgery, Barrow Neurological Institute, St. Joseph's Hospital and Medical Center, Phoenix, AZ, United States; ^2^Department of Neurosurgery, University of Arizona, Tucson, AZ, United States

**Keywords:** europium, fluorescein, fluorescence-guided tumor surgery, fluorophores, high-grade glioma, indocyanine green, protoporphyrin IX, tumor model

## Abstract

**Objective:** Fluorescence dyes are increasingly used in brain tumor surgeries, and thus the development of simulation models is important for teaching neurosurgery trainees how to perform fluorescence-guided operations. We aimed to create a tumor model for fluorescence-guided surgery in high-grade glioma (HGG).

**Methods:** The tumor model was generated by the following steps: creating a tumor gel with a similar consistency to HGG, selecting fluorophores at optimal concentrations with realistic color, mixing the fluorophores with tumor gel, injecting the gel into fresh pig/sheep brain, and testing resection of the tumor model under a fluorescence microscope. The optimal tumor gel was selected among different combinations of agar and gelatin. The fluorophores included fluorescein, indocyanine green (ICG), europium, chlorin e6 (Ce6), and protoporphyrin IX (PpIX). The tumor model was tested by neurosurgeons and neurosurgery trainees, and a survey was used to assess the validity of the model. In addition, the photobleaching phenomenon was studied to evaluate its influence on fluorescence detection.

**Results:** The best tumor gel formula in terms of consistency and tactile response was created using 100 mL water at 100°C, 0.5 g of agar, and 3 g of gelatin mixed thoroughly for 3 min. An additional 1 g of agar was added when the tumor gel cooled to 50°C. The optimal fluorophore concentration ranges were fluorescein 1.9 × 10^−4^ to 3.8 × 10^−4^ mg/mL, ICG 4.9 × 10^−3^ to 9.8 × 10^−3^ mg/mL, europium 7.0 × 10^−2^ to 1.4 × 10^−1^ mg/mL, Ce6 2.2 × 10^−3^ to 4.4 × 10^−3^ mg/mL, and PpIX 1.8 × 10^−2^ to 3.5 × 10^−2^ mg/mL. No statistical differences among fluorophores were found for face validity, content validity, and fluorophore preference. Europium, ICG, and fluorescein were shown to be relatively stable during photobleaching experiments, while chlorin e6 and PpIX had lower stability.

**Conclusions:** The model can efficiently highlight the “tumor” with 3 different colors—green, yellow, or infrared green with color overlay. These models showed high face and content validity, although there was no significant difference among the models regarding the degree of simulation and training effectiveness. They are useful educational tools for teaching the key concepts of intra-axial tumor resection techniques, such as subpial dissection and nuances of fluorescence-guided surgery.

## Introduction

Neurosurgery has entered a new era with the application of fluorescence guidance technologies, which are widely applicable in multiple surgical disciplines. To achieve maximal resection, several fluorophores have been introduced into tumor surgery.

1. Fluorescein is a fluorescent dye with an excitation wavelength range of 460–500 nm and emission range of 540–690 nm. It can be visualized under the Yellow 560 filter of the operating microscope. Fluorescein has been used to improve the resection of malignant gliomas by targeting the tumor and margins with doses ranging from 2 to 20 mg/kg administered intravenously (1–3).
2. Indocyanine green (ICG) is widely used in vascular neurosurgery to identify and evaluate the vascular pattern, but it has also been also used for malignant glioma surgeries. It has a peak excitation at a wavelength of 750–800 nm and is visualized by near-infrared cameras at an emission maximum of 850 nm. The usual administration of ICG for vascular application is an intravenous bolus of 5–25 mg, with some tumors remaining fluorescent for about 10 min (4–6). Higher doses of ICG (5 mg/kg) imaged with more sensitive cameras have been used in fluorescence-guided brain tumor resection (6, 7).
3. Protoporphyrin IX (PpIX) is produced from the bioconversion of 5-aminolevulinic acid (5-ALA), which is administered orally at doses of 10–50 mg 4 h before surgery (8). PpIX accumulates in high-grade glioma (HGG) cells and demonstrates red fluorescence with peak emission around 630 nm under blue light (405 nm) excitation, which allows for tumor cells targeted by fluorescence (9–14).
4. Chlorin e6 (Ce6) is a red fluorescent dye, a second-generation photosensitizer, which can be excited with blue light and has an absorption peak of 400 nm. It can be used to target malignant brains tumors or for photodynamic therapy with a dose of 40 mg/m^2^ body surface (15–18). Ce6 can be used for fluorescence guidance, but, unlike 5-ALA, its mechanism is not related to bioconversion; rather, similar to fluorescein and ICG, it is related to passive accumulation in the tumor (16, 19, 20).
5. Europium is a photoluminescent lanthanide that emits red light under blue light excitation. It is highly stable and may be used to target tumors in nanoparticles, which can be used in experimental models (21–24). We have demonstrated that europium-based materials have high photostability and can be used for calibration and normalization of quantitative PpIX fluorescence (9).

It is increasingly important to develop simulative models to educate neurosurgical residents and trainees and to help them develop surgical skills in a safe environment. However, for tumor models, it is challenging to construct a model that can mimic the intra-axial tumor resection process under fluorescence guidance in a laboratory environment. Such teaching models help the trainee to understand the key steps and nuances in fluorescence-assisted tumor resection to achieve maximal resection, especially in the case of invasive tumors, such as malignant gliomas.

In this study, we developed a “tumor gel” based on different combinations of gelatin and agar to mimic the consistency of HGG. Different fluorescent dyes were also tested to simulate the visible fluorescence under microscope magnification. We also built a training model by injecting the tumor gel into fresh sheep/pig brains to simulate a realistic tumor resection (25, 26).

## Materials and Methods

The development of this model involved two major components: the tumor gel for the consistency of the tumor and the dye for the fluorescent effect. Additionally, we tested the photobleaching effect in the models. Finally, we injected the combination of tumor gel and fluorescent agents into fresh sheep and pig brains and tested the model among neurosurgeons and neurosurgical trainees using a scoring system.

### Tumor Gel

A tumor gel was developed based on different combinations of agar (Landor Trading Co. Agar Powder) and gelatin (Knox Original Gelatin, Unflavored) to simulate the consistency and firmness of HGG.

**Figure 1:**
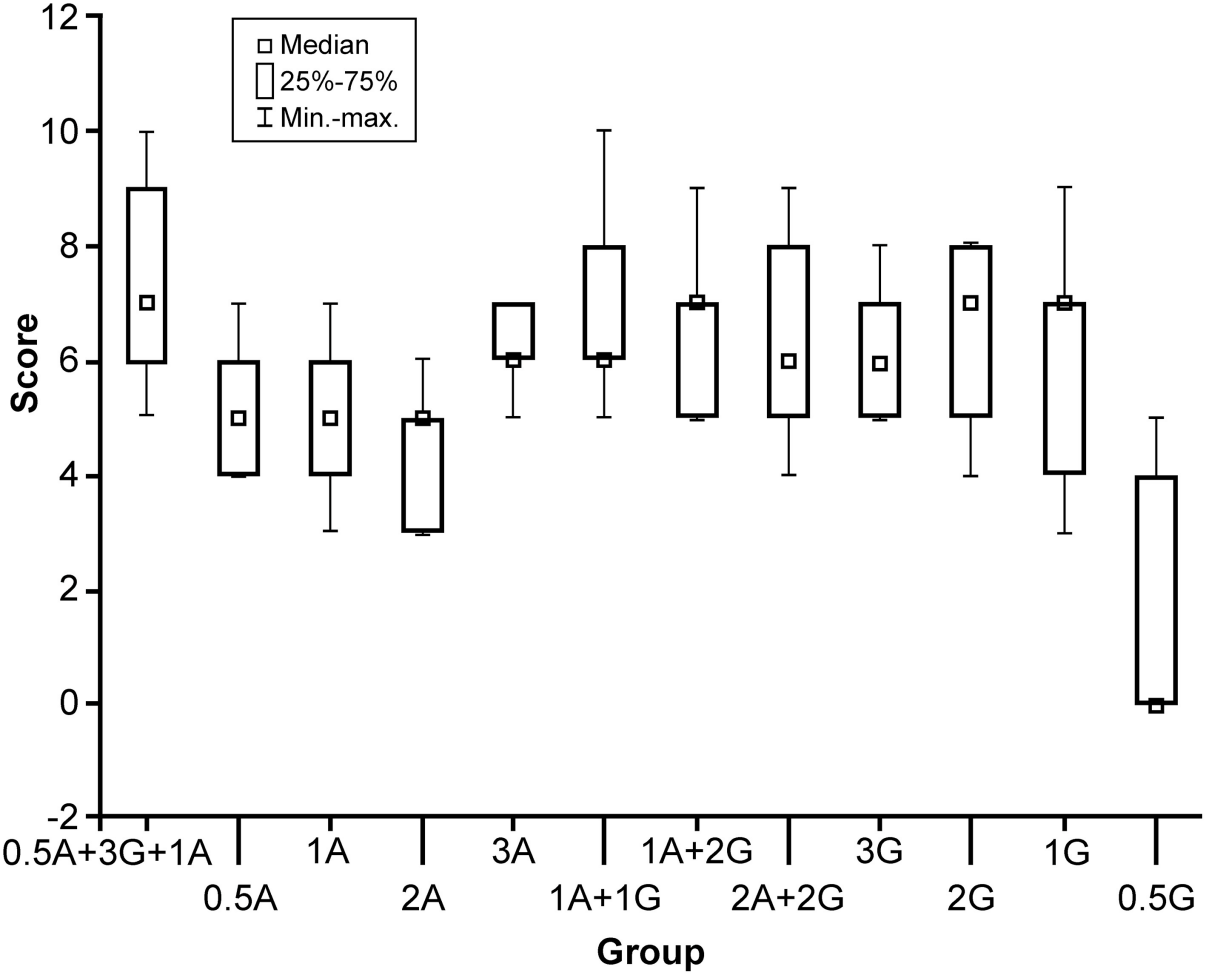
Box plot comparison of 12 solutions with different formulas (horizontal axis) and their scores (0–10, vertical axis). The formula with the highest score was 0.5 g agar and 3 g gelatin with 100 mL boiling water, with additional 1 g of Agar added at 50°C. A, agar; G, gelatin; min-max, minimum to maximum values. *Used with permission from Barrow Neurological Institute, Phoenix, Arizona*.

A preliminary experiment was performed to find the optimal proportions of agar and gelatin by mixing different amounts with 100 mL of boiling water. Agar and gelatin were tested separately with amounts of 0.2, 0.3, 0.4, 0.5, and 1–5 g in 0.5 g increments and together in various proportions (Figure 1). The powder was dissolved thoroughly in boiling water for 3 min to generate a homogeneous polymer.

After elimination of clearly unworkable combinations (too soft or too hard), 12 different combinations of agar or gelatin or both were included in subsequent tests. Six neurosurgeons with broad experience in brain tumor surgery tested and scored all 12 combinations, using a scoring system from 0 to 10 (10 meant the best simulation of an HGG). Equipment and instruments used to assess the gels included those that would normally be used for resecting an invasive brain tumor, such as operating microscope, bipolar forceps, microdissectors, microsuction, microscissors, and microforceps.

To increase the granulation to mimic a real tumor, an additional 1 g of agar was added to the tumor gel (100 mL solution) during cooling (at 50°C).

### Optimal Concentrations of Fluorescent Agents

We acquired images of the following dyes at different concentrations: fluorescein (AK-FLUOR Fluorescein Injection, USP), ICG (Cardiogreen Sigma-Aldrich I2633), europium (Sigma-Aldrich), Ce6 (Frontier Scientific), and PpIX (free acid, Enzo Life Sciences). Serial dilutions of the fluorescent dyes were performed to obtain solutions at different concentrations. Normal saline was used as a control for fluorescein and ICG, and dimethyl sulfoxide was used as a control for the remaining three fluorophores. Fluorescein was diluted into 24 shares with concentrations ranging from 1.5 × 10^−6^ to 12.5 mg/mL. ICG was diluted into 17 shares with concentrations ranging from 3.1 × 10^−4^ to 10 mg/mL. Europium was diluted into 18 shares with concentrations ranging from 5.5 × 10^−4^ to 36 mg/mL. Ce6 was diluted into 23 shares with concentrations ranging from 8.6 × 10^−6^ to 36 mg/mL. PpIX was diluted into 22 shares with concentrations ranging from 1.7 × 10^−5^ to 36 mg/mL.

Images were captured with an operating microscope (Kinevo 900 OPMI, Carl Zeiss, Oberkochen, Germany) and a camera attached to the observing ocular lens of the microscope (Cannon EOS Rebel T2i, Tokyo, Japan). Different filters of the operating microscope were used with the filters: Blue 400 was used in the analysis of europium, Ce6, and PpIX; Yellow 560 for fluorescein; and Infrared 800 with and without overlay for ICG. A long-wave pass (LWP) filter (B+W F-Pro 58 090 5 × E, Schneider Kreuznach, Bad Kreuznach, Germany) was attached to the external camera to accurately measure and analyze PpIX, Ce6, and europium fluorescence.

Settings of the microscope were focus 5 × and distance 200 mm, with additional light used when applicable (Blue 400 and Yellow 560), and 1/15 s shutter speed (for the microscope's internal camera) for all images. Procedures using ICG were performed at 25% light intensity (68 mW/cm^2^), and the procedures using fluorescein were performed using a Yellow 560 filter at 20% light intensity (9.7 mW/cm^2^). The external camera was set at 1/4 s exposure and light sensitivity of the imaging sensor of ISO (International Organization for Standartization) 100 for ICG and fluorescein. Procedures using europium, Ce6, and PpIX were performed under a Blue 400 filter at 100% light intensity (13.4 mW/cm^2^), with the red filter attached on the external camera set to 4/5 s exposure and ISO 400. Irradiance was measured using an S120VC photodiode power sensor and a PM400 power meter calibrated to 405 nm for Blue 400, 532 nm for Yellow 560, and 785 nm for Infrared 800 mode (Thorlabs, Dachau, Germany) (9).

Optimal dye concentrations used in the preparation of the fluorescent tumor gels (FTG) were selected by comparing the colors directly under the ocular lens of the microscope. Images were then analyzed in Fiji (27) using the mean pixel signal intensity of the region of interest to confirm the relationship between the relative fluorescence intensity and concentration to select the optimal concentration. Multichannel analysis was used for ICG and single-channel analysis in the others—the green channel for fluorescein and the red channel for europium, Ce6, and PpIX.

### Fluorescent Tumor Gel Preparation

The FTG was created by mixing the selected fluorescent dye with the tumor gel immediately after the addition of booster agar at 50°C (see above). To generate the FTG, 5 mL of tumor gel was mixed with each dye at previously selected concentrations.

### Photobleaching

A known phenomenon that may affect the interpretation of fluorescence during a period of continuous observation, photobleaching was taken into account and compared among the different FTGs.

Different types of FTG were tested under microscope light with corresponding filters with continuous observation for 5 min, which corresponds to the clinically expected duration of the imaging before altering the microscope position. The photobleaching effect in ICG was recorded using ICG angiography mode as videos, whereas for the remainder of the dyes, a series of images was acquired every 6 s using the external camera. Frames of the ICG video were extracted as images every 6 s using GOM Lab (Gom & Company, South Korea). The settings of the microscope, filter, and camera were the same as above.

The light intensity of each image was extracted during the 5-min continuous observation using Fiji in the region of interest with the same methods as described above.

### Tumor Model Preparation

Cooled sheep and pig brains, purchased from a local meat source, were used for the final tumor model preparation to simulate brain tumor resection. A total of 5 mL FTG solution (5 mL FTG and dye at its corresponding concentration) was aspirated in a 5 mL syringe attached to a 21-gauge needle. The FTG was subsequently injected into a fresh sheep and pig brain subcortically; only 1.5–2 mL of the FTG was injected because the size of sheep and pig brain is less than the size of a human brain. The injection was performed meticulously to avoid leakage using a previously described technique (28).

### Tumor Model Resection

Three fully trained neurosurgeons and 2 neurosurgical residents (PGY1 and PGY6) assessed the tumor model. Their general information and experience of neurosurgery were collected. They performed simulative tumor resection under microscopic magnification (Kinevo 900 OPMI, Carl Zeiss, Oberkochen, Germany) with and without the assistance of fluorescence guidance. The participants were instructed to perform subpial dissections and to preserve the normal brain tissue. All FTGs were tested by all participants under the corresponding microscopic filters. The simulative operative procedure included operating microscope, and instruments including bipolar forceps, microdissectors, microsuction, microscissors, and microforceps.

### Questionnaire of Satisfaction About the Tumor Model

Participants evaluated the model using a questionnaire with items about the face validity, content validity, and white light comparison. Face validity questions evaluate the realism of the model, whereas content validity is a qualitative measure of the appropriateness of the model as a teaching modality (29). The answering system was structured with scores 1–5, in which 1 means “strongly disagree” and 5 means “strongly agree” for all but one question, in which 1 means “easier” and 5 means “more difficult.” The questionnaire is shown in Supplemental Figure 1.

## Results

### Tumor Gel

Results of the comparison of all tumor gel formulas are illustrated in Figure 1. The best tumor gel formula in terms of consistency and tactile response similar to HGG was the first formula in Figure 1: 100 mL 100°C water, 0.5 g of agar, and 3 g of gelatin mixed thoroughly for 3 min (P_Kruskal−Wallis_ = 0.048). One gram of agar was added when the tumor gel was at 50°C during the cooling down period.

### Optimal Concentrations of Fluorescent Agents

**Figure 2:**
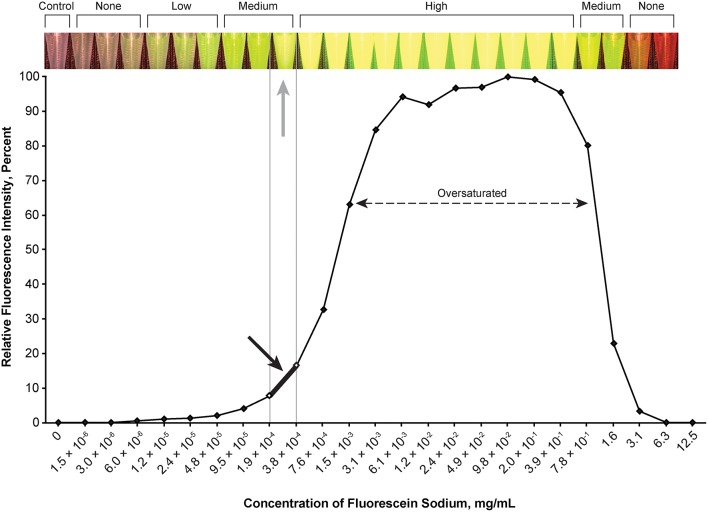
Relationship of relative fluorescence intensity and different concentrations of fluorescein as recorded by the camera. The fluorescence with the concentration ranging from 6.1 × 10^−3^ mg/mL to ~4.0 mg/mL is oversaturated. The heading bar shows the fluorescence color and intensity at different concentrations (*gray arrow* indicates optimal intensity). A concentration of 1.9 × 10^−4^ to 3.8 × 10^−4^ mg/mL was subjectively selected as the optimal concentration for the tumor model (*black arrow*) by observing the color. *Used with permission from Barrow Neurological Institute, Phoenix, Arizona*.

**Figure 3:**
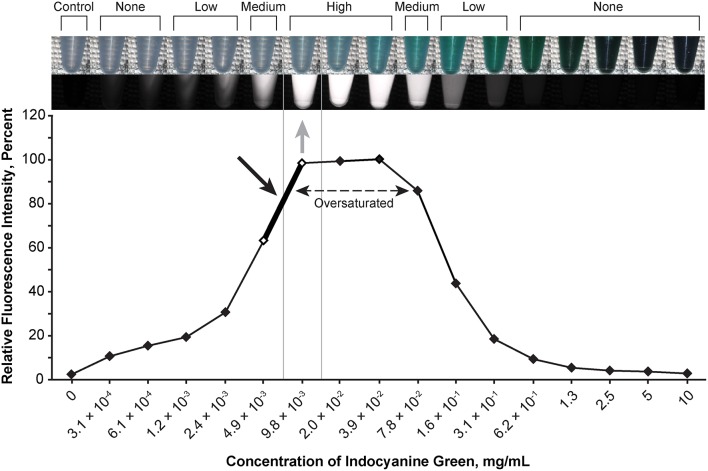
Relationship of the relative fluorescence intensity and different concentrations of indocyanine green as recorded by the camera. The fluorescence with the concentration ranging from 2.0 × 10^−2^ mg/mL to 3.9 × 10^−2^ mg/mL is oversaturated. The heading bar shows the fluorescence color and intensity at different concentrations (*gray arrow* indicates optimal intensity). A concentration of 4.9 × 10^−3^ to 9.8 × 10^−3^ mg/mL was subjectively selected as the optimal concentration for the tumor model (*black arrow*) by observing the color. *Used with permission from Barrow Neurological Institute, Phoenix, Arizona*.

**Figure 4:**
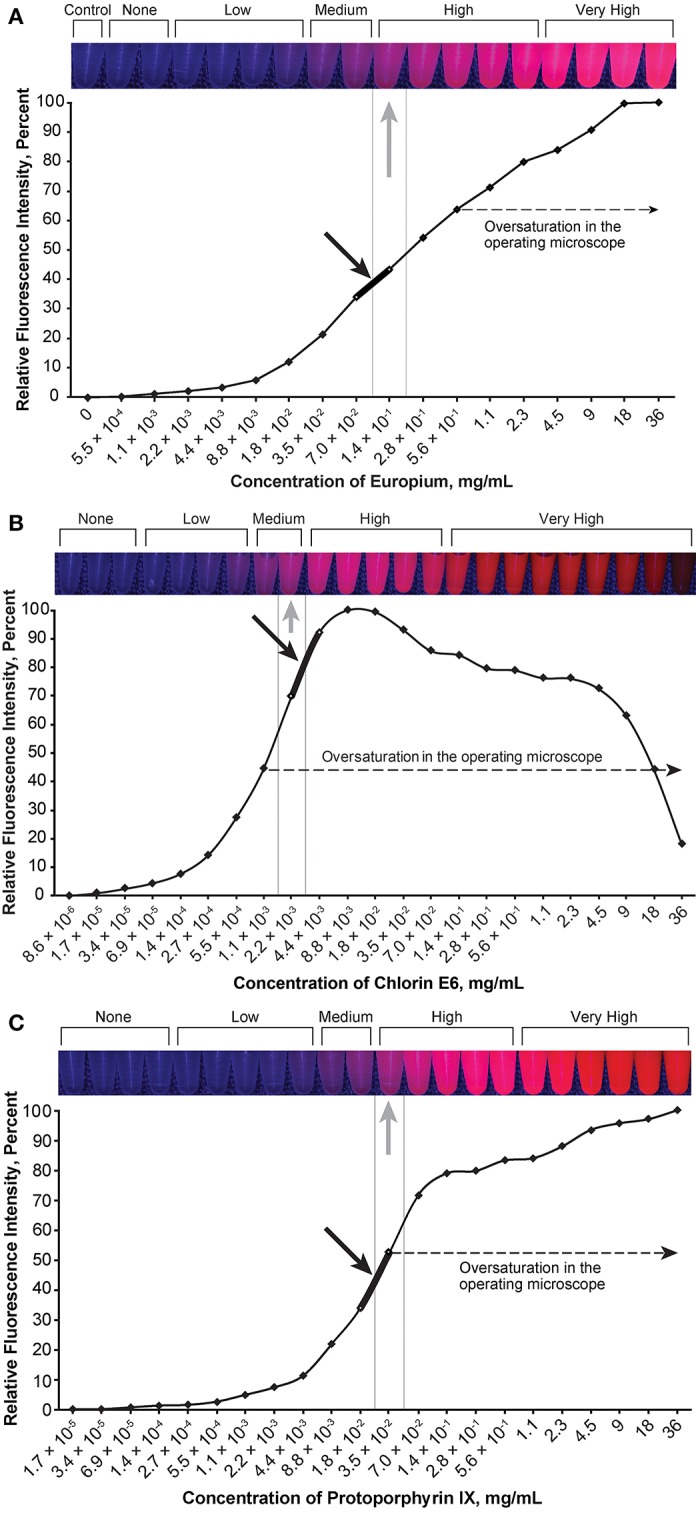
Relationship of the relative fluorescence intensity and different concentrations of europium **(A)**, chlorin e6 **(B)**, and protoporphyrin IX **(C)**. The heading bars show the fluorescence color and intensity at different concentrations (*gray arrows* indicate optimal intensity). For europium **(A)**, the fluorescence was oversaturated when the concentration was higher than 5.6 × 10^−1^ mg/mL, and the concentration range of 7.0 × 10^−2^ to 1.4 × 10^−1^ mg/mL was subjectively selected as the optimal concentration for the tumor model (*black arrow*) by observing the color. For chlorin e6 **(B)**, the fluorescence was oversaturated as recorded through the operating microscope camera when the concentration was above 1.1 × 10^−3^ mg/mL, and the concentration range of 2.2 × 10^−3^ to 4.4 × 10^−3^ mg/mL was subjectively selected as the optimal concentration for the tumor model (*black arrow*) by observing the color. For protoporphyrin IX **(C)**, the fluorescence was oversaturated as recorded by the microscope camera when the concentration was higher than 3.5 × 10^−2^ mg/mL, and the concentration range of 1.8 × 10^−2^ to 3.5 × 10^−2^ mg/mL was subjectively selected as the optimal concentration for the tumor model (*black arrow*) by observing the color. *Used with permission from Barrow Neurological Institute, Phoenix, Arizona*.

The optimal concentration range of fluorescein was 1.9 × 10^−4^ to 3.8 × 10^−4^ mg/mL (Figure 2), of ICG was 4.9 × 10^−3^ to 9.8 × 10^−3^ mg/mL (Figure 3), of europium was 7.0 × 10^−2^ to 1.4 × 10^−1^ mg/mL (Figure 4A), of Ce6 was 2.2 × 10^−3^ to 4.4 × 10^−3^ mg/mL (Figure 4B), and of PpIX was 1.8 × 10^−2^ to 3.5 × 10^−2^ mg/mL (Figure 4C).

During observation of ICG, as the concentration of ICG increased, the fluorescent intensity increased to oversaturation at first, and then decreased as the concentration increased. Two different concentrations produced similar optimal intensity (Figure 3), but the solution with a lower concentration was selected for convenience (ICG 4.9 × 10^−3^ to 9.8 × 10^−3^ mg/mL).

Because fluorescence intensity was lower in the agar solution than in the normal saline or dimethyl sulfoxide, the following concentrations were selected to create the FTGs: FNa-TG at the concentration of 2.44 × 10^−4^ mg/mL, ICG-TG 2.5 × 10^−2^ mg/mL, Eu-TG 1.44 × 10^−1^ mg/mL, and Ce6-TG 1.125 × 10^−2^ mg/mL. These FTGs were used to test the effect of photobleaching.

PpIX demonstrated unexpected behavior when mixed with the tumor gel—the red fluorescence disappeared very quickly, with the gel appearing somewhat brown-black. Hydrochloric acid (12M) was added to the FTG in a 1:10 ratio before the dye was mixed (PpIX, 1.1 × 10^−3^ mg/mL) to circumvent this behavior based on the pH-sensitive property of 5-ALA (30), which eventually prevented the color change.

### Photobleaching

**Figure 5:**
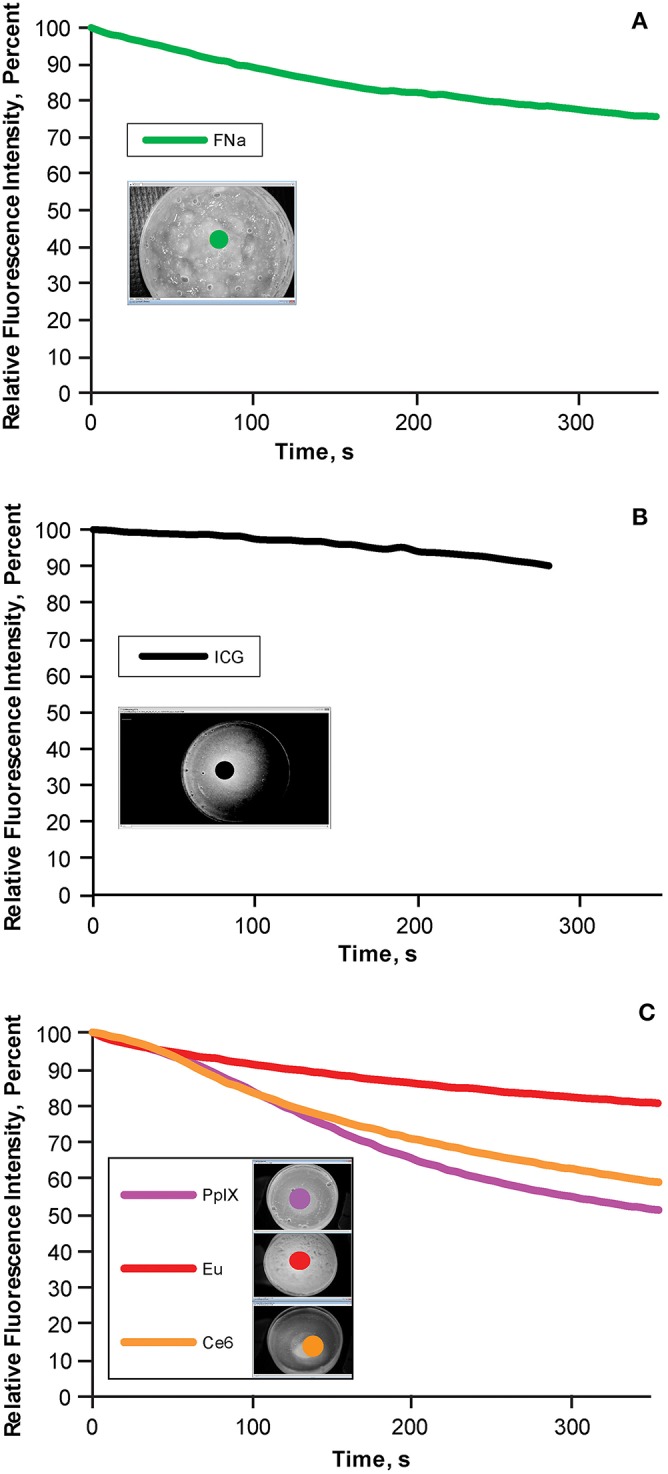
Bleaching phenomena: brightness decrease in different fluorescent tumor gels. **(A)** Fluorescein (FNa); **(B)** indocyanine green (ICG); and **(C)** protoporphyrin IX (PpIX), europium (Eu), and chlorin e6 (Ce6), which are demonstrated together because the same filter was used for all three. During the 5-min (300-s) observation, the decrease in relative fluorescence intensity was approximately 20% for fluorescein, 10% for ICG, 45% for PpIX, 10% for europium, and 35% for Ce6. Inset photographs in each graph show the regions of interests (indicated by colored dots) used to quantify the fluorescence intensity over time. *Used with permission from Barrow Neurological Institute, Phoenix, Arizona*.

During the 5-min photobleaching test, we observed decreases of 22.3% in fluorescein (Figure 5A), 10% in ICG (Figure 5B), 17.57% in europium (Figure 5C, red), 37.56% in Ce6 (Figure 5C, orange), and 44.84% in PpIX (Figure 5C, light purple). The rate of bleaching effect was higher for PpIX than the rates for europium and Ce6.

### Tumor Model Resection Test

**Figure 6:**
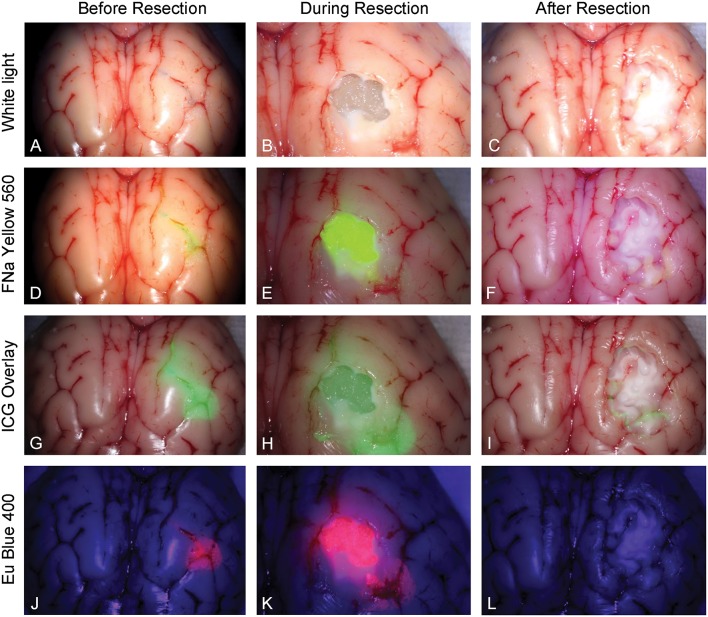
Tumor model in a pig brain at different stages of resection viewed under white light and with the various fluorophores (fluorescein [FNa], indocyanine green [ICG], and europium [Eu]) viewed under their corresponding filters. Tumor model viewed under white light **(A)** before, **(B)** during, and **(C)** after resection. Tumor model after application of fluorescein **(D)** before, **(E)** during, and **(F)** after resection under Yellow 560 filter. Tumor model after application of ICG **(G)** before, **(H)** during, and **(I)** after resection in the ICG overlay mode. Tumor model after application of europium **(J)** before, **(K)** during, and **(L)** after resection under Blue 400 filter. *Used with permission from Barrow Neurological Institute, Phoenix, Arizona*.

Five neurosurgeons and neurosurgical trainees aged 28–37 years tested the tumor model. Every participant performed 5 resections (1 operation per fluorophore). Three out of 5 had at least 2 years of experience in microneurosurgery including glioma resection experience. None had previous experience with any tumor model for resection training. The tumor models under different stages of resection and under different fluorescent modes are shown in Figure 6.

### Face Validity

**Figure 7:**
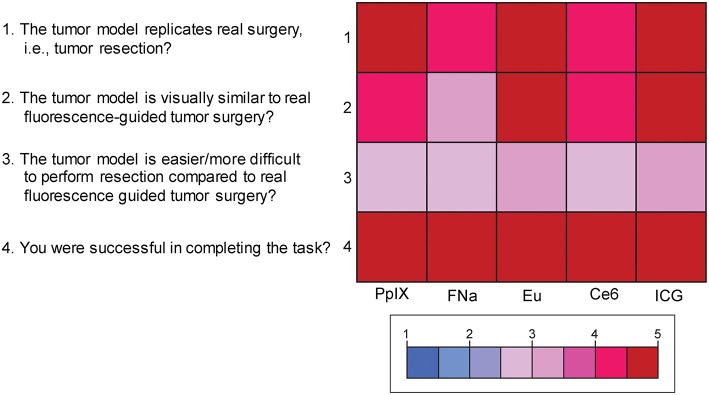
Color-coded plot for questions about face validity. The colored bar shows the scores and their corresponding colors; the score for each question was the average score based on the questionnaire. *Used with permission from Barrow Neurological Institute, Phoenix, Arizona*.

Mean scores of the questions for face validity are shown in Figure 7. On average, participants answered with a high degree of agreement on the first four questions, which indicates high face validity of the developed model. There were no significant differences among the different dyes for each individual face validity question or for the sum of the four scores (P_Kruskal−Wallis_ > 0.05 for all, see Supplemental Figures 2, 3).

### Content Validity

**Figure 8:**
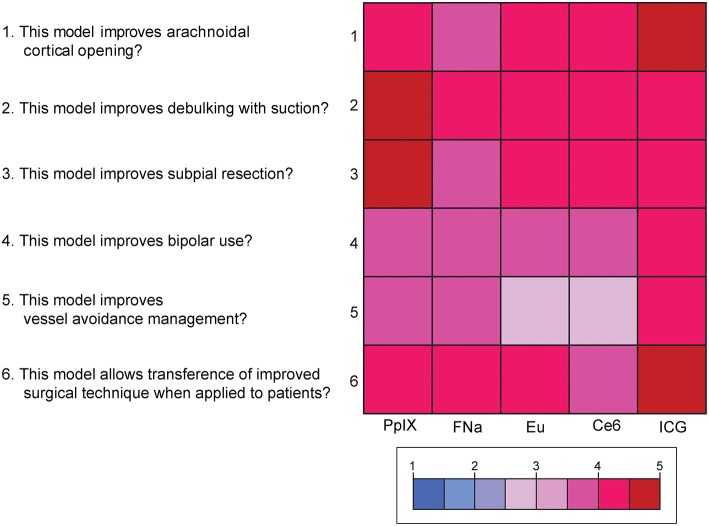
Color-coded plot for questions about content validity. The colored bar shows the scores and their corresponding colors; the score for each question was the average score based on the questionnaire. *Used with permission from Barrow Neurological Institute, Phoenix, Arizona*.

Mean scores for the questions for content validity are shown in Figure 8. On average, participants answered with a high degree of agreement on the six questions regarding the content validity of the model, which indicates high content validity of the developed model for the purpose of simulating fluorescence-guided surgical tumor resection. There were no significant differences among the different dyes for each individual content validity question or for the sum of the four scores (P_Kruskal−Wallis_ > 0.05 for all, see Supplemental Figure 4).

### Fluorophores Preferences

**Figure 9:**
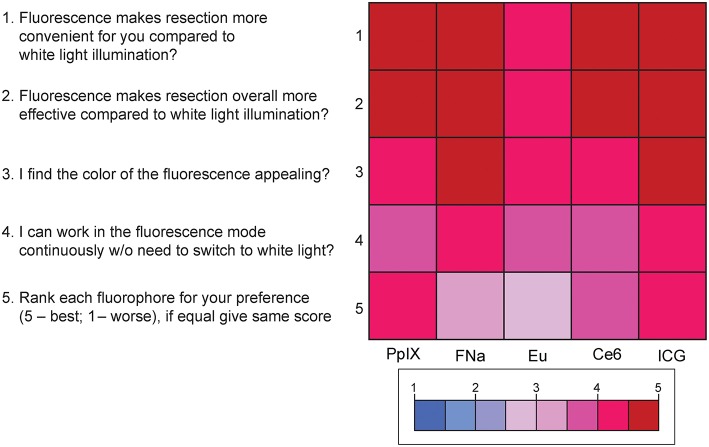
Color-coded plot for questions about fluorophore preferences. The colored bar shows the scores and their corresponding colors; the score for each question was the average score based on the questionnaire. *Used with permission from Barrow Neurological Institute, Phoenix, Arizona*.

Results of the subjective comparative evaluation between the fluorophores are shown in Figure 9. On average, participants answered with a high degree of agreement on the questions regarding the convenience, effectiveness, and visual appeal of the fluorescence-guidance of the model. Moreover, when ranking the fluorophores, no fluorophore was significantly better or worse than the others. There were no significant differences among the different dyes for each of the final five questions (P_Kruskal−Wallis_ > 0.05 for all, see Supplemental Figure 5), indicating that there was no fluorophore that was subjectively superior or inferior to others among the participants.

## Discussion

For educational purposes, it is of paramount importance to learn and practice tumor resection prior to a real surgery. Length of survival has been correlated to extent and volume of tumor resection for HGG. Fluorescence techniques add a layer of complexity interpreting tumor boundary in brain tumor surgery, especially for HGG. Previous studies have shown that realistic tumor models can be used in training neurosurgery residents to help them learn the concepts of glioma surgery and the application of fluorescence in neurosurgery (25, 26).

However, those reported models did not include all of the features, such as in real brain tissue simulation and realistic colors of fluorescence-guided tumor surgery (fluorescein, ICG, and europium) along with different filters used in real surgeries. It would arguably be more valuable to have all characteristics in one model to mimic a real neurosurgical scenario. To the best of our knowledge, this is the first report of an in-brain tumor model resembling HGG combined with the use of fluorescence. The tumor model contains 3 parts: the tumor gel, which can offer a tactile response similar to a real tumor during the resection; fluorescent dyes, which can mimic the realistic application of fluorescence; and the actual sheep or pig brains. The FTG was injected in the pig and sheep brains to create a simulation model that replicates the properties of HGG and surrounding brain tissue in a human brain.

### Consistency of the Tumor Gel

A tumor model should offer realistic tactile feedback when appropriate instruments are used to help the trainees acquire a simulative experience. We developed a tumor model to simulate HGG. The consistency of HGG may vary from firm to gelatinous and, in general, demonstrates a degree of firmness between normal parenchyma and a firmer, harder metastatic lesion. During the preparation of our tumor gel formula, we created different types of tumor gels of different firmness and consistencies, including those that resemble the metastatic lesions (the solutions of 3 g agar in 100 mL water or 2 g agar plus 2 g gelatin in 100 mL water). The latter recipe results in a slightly firmer, more solid consistency that more closely resembles a metastatic lesion, rather than an invading glioma. During preparation of the gel, we also noticed that the thorough mixture of all ingredients in 100°C water produced a tumor gel that was highly homogeneous, whereas a real glioma would be more granulated. In order to create the granularity, we added a booster dose of agar at 50°C during the cooling-down period.

During any intra-axial brain tumor resection, identifying the margin of the tumor and working in the plane between the tumor and normal parenchyma is almost always the principle goal. This technique requires the tumor model to have a similar, but not the same, consistency as normal parenchyma. The margin of the tumor model is easy to locate, especially with the assistance of fluorescence. This goal may be realistic in HGG or metastatic tumors; however, low-grade glioma usually does not have a clear plane of margin, and fluorescence techniques to identify tumor tissue do not appear to work well in such tumors. Our model is not able to mimic the resection of low-grade glioma, which is one of the limitations of the study.

### Suitable Color of Fluorescence

A suitable color shown in the tumor model can realistically mimic the fluorescence used in fluorescence-guided surgery. Obtaining a suitable color requires choosing the optimal concentration of each fluorophore. In our study, a series of dilutions were performed to obtain solutions with different levels of concentration. In our experiment, all fluorophores successfully displayed adequate color after mixing with the tumor gel except for PpIX, which had an unexpected behavior. After mixing, the fluorescence of PpIX vanished quickly and the tumor gel turned dark brown. PpIX is an acidophilic substance, which may not work properly in the basic environment of the tumor gel (30). When hydrochloric acid was added into the tumor gel to acidify the tumor gel before adding PpIX, the gel was fluorescent and used in the resection; however, this extra step makes the preparation of PpIX FTG cumbersome, and hydrochloric acid can be dangerous to handle.

The concentrations of fluorophores chosen in our study were selected specifically for application and their functionality with the tumor gels and simulation model development. These concentrations do not represent the concentration of the fluorescent drugs used in actual surgery and accumulated in the tumor tissue, as multiple environmental factors, such as pH, protein binding, and others, affect actual fluorescence intensity in patients. Additionally, brain tumors, especially HGG, are often highly heterogeneous in their cytoarchitecture and present with varying levels of fluorescence intensity within the tumor (31–33).

### Advantages and Disadvantages of Fluorophores

Fluorescence techniques are widely used in neurosurgical subdisciplines, and learning to operate under fluorescence is increasingly important for neurosurgeons. The different fluorophores have advantages and disadvantages, and the features of each fluorophore should be well-understood by any neurosurgeon using fluorescent techniques. Recent excellent reviews discuss fundamental aspects of fluorescence-guided surgery in greater detail (34–36).

Fluorescein is a bright yellow fluorophore used off-label for tumor labeling in neurosurgery, with a growing body of evidence suggesting that its use improves achievement of gross total resection (37–39). Advanced illumination capabilities of the latest operating microscopes allow neurosurgeons to work directly under the Yellow 560 filter throughout the tumor resection (40).

ICG is used in vascular neurosurgery to evaluate the patterns of the vasculature (41), and recent studies suggest its feasibility for brain tumor fluorescence-guided surgery (42). New operating microscopes provide the function of real-time ICG overlay (Figures 6G–I), in which the ICG signal is injected into the original white light optical pathway as a semitransparent green overlay (43–45). This enables undistracted continuous manipulations and observation of both normal anatomy and ICG signal, which would be ideal for fluorescence-guided surgery of brain tumors. ICG allows for subsurface imaging as demonstrated by Figure 6H. Additionally, there was a tiny rim of residual ICG signal (Figure 6I), which represents staining of the pia. Whether this finding represents better sensitivity in detecting ICG signal compared with other fluorophores requires further investigation.

Europium, Ce6, and PpIX are all red fluorophores that can be used for simulation models. We used europium because of its fluorescence spectral similarity to PpIX and the additional benefit that, unlike PpIX, europium is not fast bleaching and can be observed for a relatively longer period than PpIX fluorescence. Ce6 is a photosensitizer that has also been explored which has the same fluorescent color and works under the Blue 400 filter, the illumination of which is blue. Therefore, even though the tumor can be illuminated by fluorescence, it might be challenging to continuously operate under fluorescence because blood is difficult to visualize. As well, switching between the Blue 400 filter and white light illumination is imperative in real surgery. In our tumor model, there is no bleeding, thus the participants were able to perform the tumor resection under Blue 400 filter without switching, which is not realistic and is one of the major limitations of this model.

Fluorescein and 5-ALA are observed through two separate optical pathways, and therefore can be visualized in stereoscopic three-dimensional mode, which can offer the perception of depth to the observers of the operation. However, ICG, with or without the overlay, is detected through a single optical pathway, and therefore can only be visualized as a two-dimensional image or a semi-transparent pseudo-colored overlay, which lacks depth, making it disadvantageous in terms of practical and educational purposes.

Fluorescence intensity does not necessarily have a linear correlation with the concentration of the fluorophore, which necessitates careful dose selection. With increased concentration, after reaching a peak of fluorescence intensity, all fluorophores are amenable to concentration-dependent quenching, the effect of decreased fluorescence when the concentration of the fluorophore is too high (35). Figures 2–6 demonstrate this concentration-dependent quenching phenomenon.

Satisfaction scores were relatively high for all the fluorophores. This could mean that, if the tumor cells are labeled with different colors, this sole difference in labeling color would not be likely to affect the efficacy of the resection.

### Significance of Photobleaching in Fluorescence Application

Photobleaching is a well-known phenomenon in which the intensity of fluorescence may decrease as the fluorophore is exposed under the excitation light during a prolonged period. This phenomenon may interfere with the interpretation of the fluorescence as the intensity is altered.

Our results demonstrated that fluorescein, ICG, and europium were relatively stable regarding the fluorescence intensity. Among the 3 fluorophores that can mimic the color of 5-ALA, europium is the most stable, which makes it a superior choice for the red fluorescence model.

### Realistic Brain Tumor Model: Significance in Practical Education

This project has a potential educational impact for less experienced neurosurgical trainees. They can practice the simulative tumor resection to understand the key concepts of intra-axial brain tumor resection. Subpial resection is a method that can maximize the resection of the lesion while minimizing the risk of damage to the nearby gyrus. It can be deleterious to practice such techniques in real situations, because beginners may not understand the difference between the consistencies of lesions and normal parenchyma. It is certainly valuable for the trainee or less experienced neurosurgeon to practice such concepts prior to the real operation (28).

Because they are hazardous materials, fluorescent dyes are not always available in a laboratory situation for practice. In the tumor model, we created a FTG, that can be shaped and injected easily. Neurosurgical beginners can learn the variations in appearance of fluorescence of different dyes at various positions and light intensities of the microscope while handling a model with a similar consistency to human tissue. The FTG can be widely used in courses because it is an affordable option, is quick and easy to prepare, and has a formula that is replicable. Based on the formula, future models of different types of brain tumors with different consistencies (i.e., metastatic tumor) can be reproduced following the same steps.

Although we did not assess the long-term stability of the FTGs, if necessary, they can be kept in air-tight bags to prevent drying. After being stored for 1 month in a light-protected place, the gels were still fluorescent; however, we believe that freshly prepared gels are better because the tumor gel may dry up, and the concentration of the fluorophore may alter during long-term preservation. Also, sodium azide can be added to the tumor gel to make a 0.01% (weight/volume) solution in order to prevent the growth of mold over time. Caution should be exercised because sodium azide is toxic.

### Limitations

The tumor model has several limitations. The tumor model does not permit the practice of skills in identifying and handling vessels; therefore, control of bleeding cannot be taught with this model. Because there is no bleeding, the participants can perform the resection under blue light without switching back to white light (blood is difficult to visualize in the dark fluorescence mode), which is not realistic and may have biased the effectiveness of 5-ALA. After resection, it is difficult to evaluate surrounding brain parenchyma damage. The tumor model cannot mimic the tumor margin of low-grade gliomas. This model cannot represent the normal human brain anatomy because both sheep and pig brains are much smaller. Additionally, assessment of the firmness and consistency of the tumor gels by 6 neurosurgeons was done subjectively. Therefore, the firmness and texture of the selected tumor gel recipe may not accurately represent HGG but can be viewed as a close approximation by neurosurgeons experienced in brain tumor surgery. Finally, the study was probably underpowered for the detection of small subjective differences among the fluorophores. The absence of significant differences among the tested fluorophores reflects the heterogeneity in the questionnaire responses regarding these fluorophores (5-ALA, fluorescein, and to a lesser extent ICG) in the neurosurgical literature (46, 47).

## Conclusion

In this paper, we present and compare 5 simulation models for fluorescence-guided brain tumor surgery, specifically of HGG. The models efficiently highlighted the “tumor” with 3 different colors—green, yellow, or infrared pseudocolored in green—and multicolor labeling is possible. These models exhibited high face and content validity, and there was no significant difference in the fluorescence-guided surgery performance or fluorescence appearance scores between the models. These models may be effective educational tools for learning the key concepts and technical nuances of fluorescence-guided brain tumor surgery associated with intra-axial tumor resection.

## Data Availability

The raw data supporting the conclusions of this manuscript will be made available by the authors, without undue reservation, to any qualified researcher.

## Author Contributions

EB and MP: conception and design of the study. DV, EB, and XZ: developed the tumor gels and assessed fluorescence measures. SG, CC, NM, PN, EB, and DV: assessed the tumor model. DV and XZ: wrote the first draft of the manuscripts. EB and DV: performed the statistical analysis. PN, ML, and MP: contributed funding, resources acquisition, and supervision. All authors contributed to manuscript revision and read and approved the submitted version.

### Conflict of Interest Statement

The authors declare that the research was conducted in the absence of any commercial or financial relationships that could be construed as a potential conflict of interest.

## Supplementary Material

Supplemental Figure 1 The questionnaire about the tumor model administered to participants. The answering system was structured with scores 1–5, in which 1 means “strongly disagree” and 5 means “strongly agree” for all but one question, in which 1 means “easier” and 5 means “more difficult.” *Used with permission from Barrow Neurological Institute, Phoenix, Arizona*.

Supplemental Figure 2 Mean scores of answers to questionnaire. *Used with permission from Barrow Neurological Institute, Phoenix, Arizona*.

Supplemental Figure 3 Scores for the 4 questions for face validity of the tumor model. Scores for the question “The tumor model replicates real surgery?” ranged from 4.4 to 4.8 (highest score was for ICG); from 4 to 5 for the question “The tumor model is visually similar to real fluorescence-guided tumor surgery?” (highest for europium [Eu]); from 2.6 to 3 (3 meaning the most adequate) for the question “The tumor model is easier/more difficult to perform resection compared to real fluorescence-guided tumor surgery?” (most adequate was ICG); and from 4.6 to 5 for the question “You were successful in completing the task?” (highest for ICG). No comparison reached statistical significance. *Used with permission from Barrow Neurological Institute, Phoenix, Arizona*.

Supplemental Figure 4 Scores for the 6 questions for content validity of the tumor model. Scores for the question “This model improves arachnoidal cortical opening?” ranged from 3.8 to 4.8 (highest score was ICG); from 4 to 4.8 for the question “This model improves debulking with suction?” (highest for ICG); from 3.8 to 4.6 for the question “This model improves subpial resection?” (highest for PpIX); from 3.6 to 4 for the question “This model improves bipolar use?” (highest for ICG); from 3.2 to 4 for the question “This model improves vessel avoidance/management?” (highest for ICG); and from 3.8 to 4.8 for the question “This model allows transference of improved surgical technique when applied to patients?” (highest for ICG). No comparison reached statistical significance. *Used with permission from Barrow Neurological Institute, Phoenix, Arizona*.

Supplemental Figure 5 Scores for the 5 questions for comparing the fluorophores. The highest and lowest scores for the question “The fluorescence makes resection more convenient for you compared to white light illumination?” were 4.8 (ICG) and 4.2 (europium [Eu]); 5 (ICG) and 4.4 (europium) for the question “The fluorescence makes resection overall more effective compared to white light illumination?”; 4.8 (fluorescein sodium [FNa] and ICG) and 4.2 (chlorin e6 and PpIX) for the question “You find the color of the fluorescence appealing?”; and 4.4 (ICG) and 3.6 (europium and chlorin e6) for the question “You can work in the fluorescence mode continuously without need to switch to white light?” For the question asking participants to rank the fluorophores in order of preference (5 being the best and 1 the worst), ICG had the highest score, followed by PpIX, Ce6, fluorescein sodium, and europium. No comparison reached statistical significance. *Used with permission from Barrow Neurological Institute, Phoenix, Arizona*.

## Abbreviations

5-ALA: 5-aminolevulinic acid
Ce6: chlorin e6
FNa: fluorescein
FTG: fluorescent tumor gel
HGG: high-grade glioma
ICG: indocyanine green
PpIX: protoporphyrin IX.
