# Supplementary figures and images for: Development of a Simulation Model for Fluorescence-Guided Brain Tumor Surgery
Source: Front Oncol. 2019 Aug 16;9:748. doi: 10.3389/fonc.2019.00748 (PMC6706957; doi:10.3389/fonc.2019.00748)

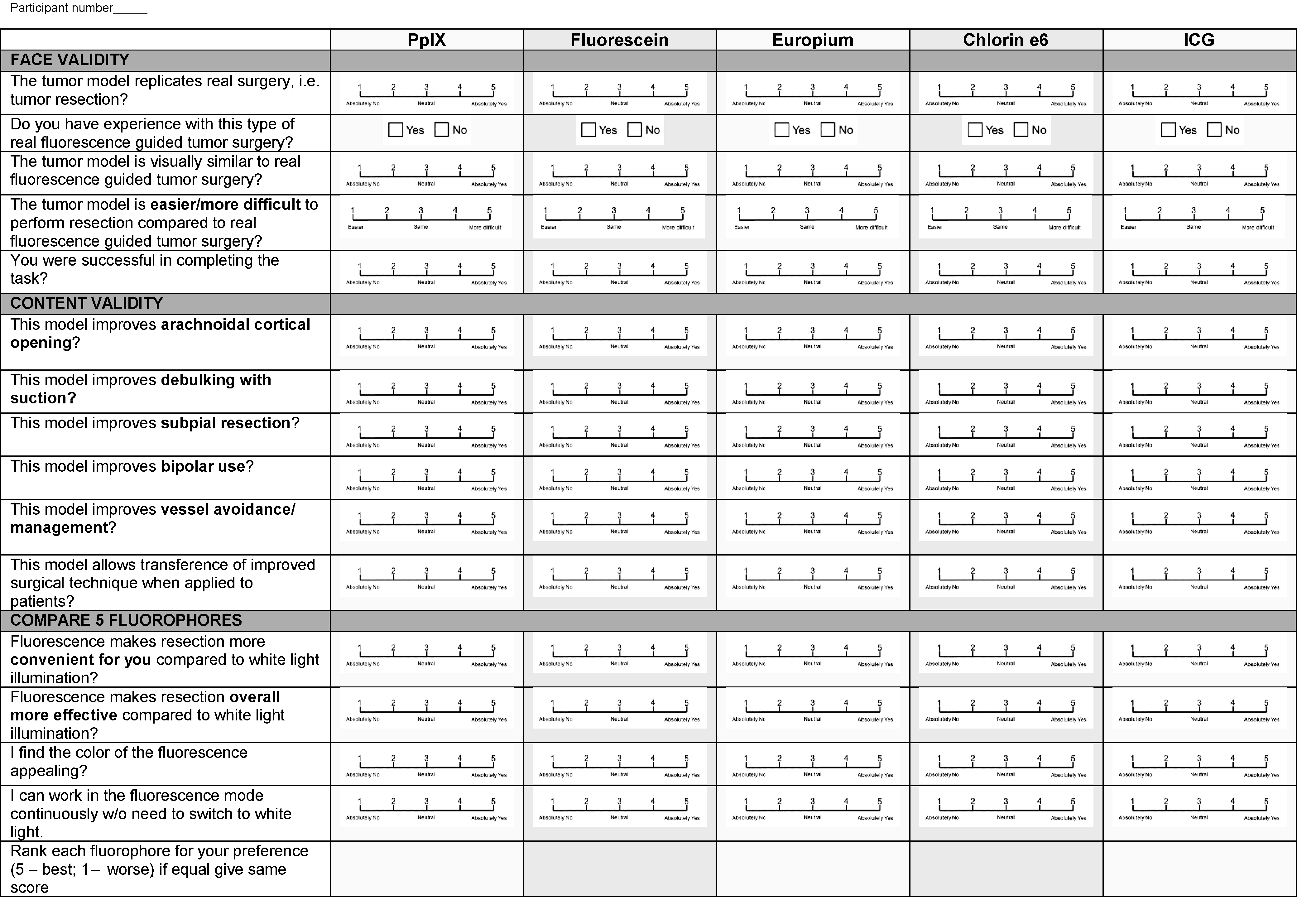

Supplement: Supplemental Figure 1 — The questionnaire about the tumor model administered to participants. The answering system was structured with scores 1–5, in which 1 means “strongly disagree” and 5 means “strongly agree” for all but one question, in which 1 means “easier” and 5 means “more difficult.” Used with permission from Barrow Neurological Institute, Phoenix, Arizona. [file Image_1.TIF]

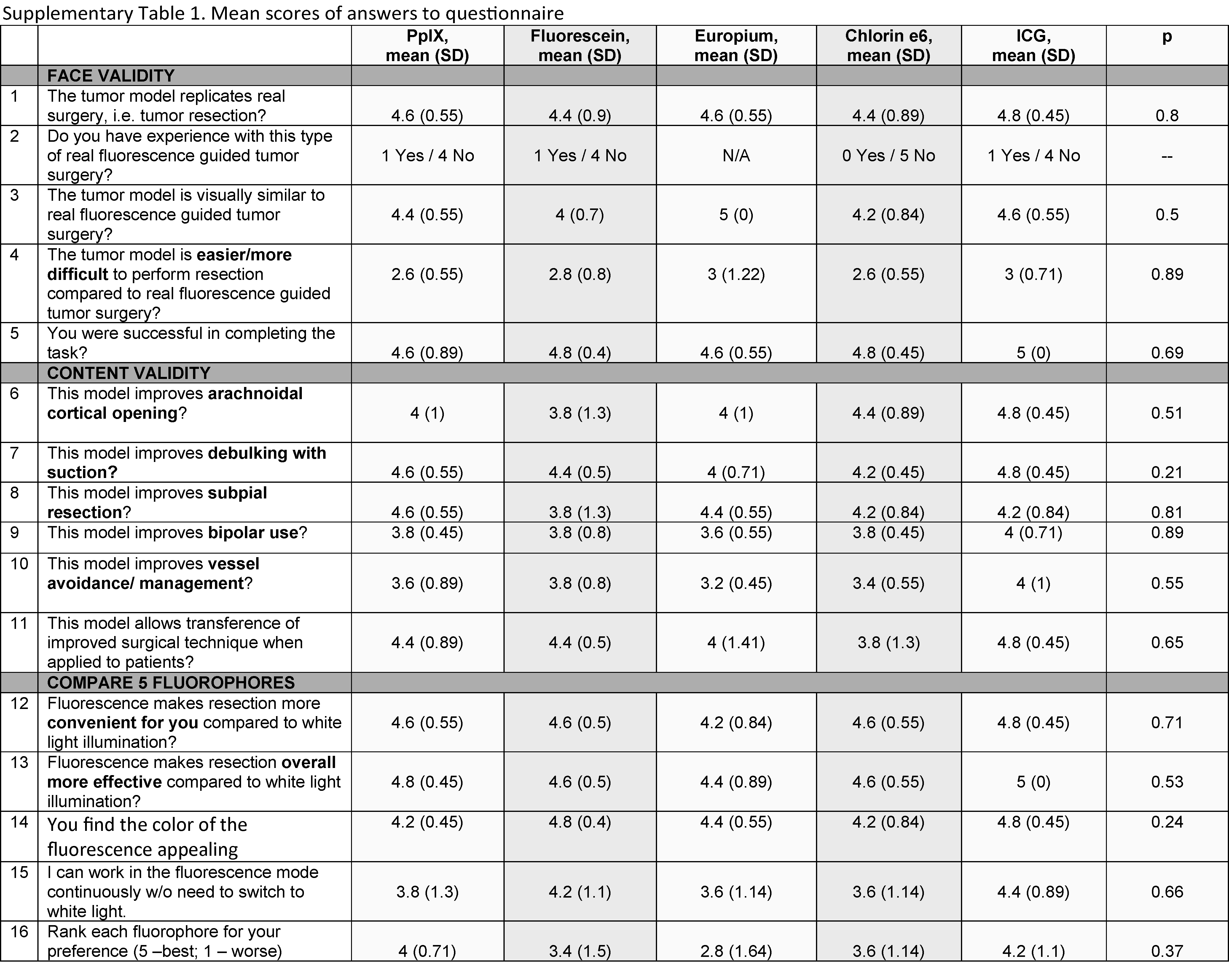

Supplement: Supplemental Figure 2 — Mean scores of answers to questionnaire. Used with permission from Barrow Neurological Institute, Phoenix, Arizona. [file Image_2.TIF]

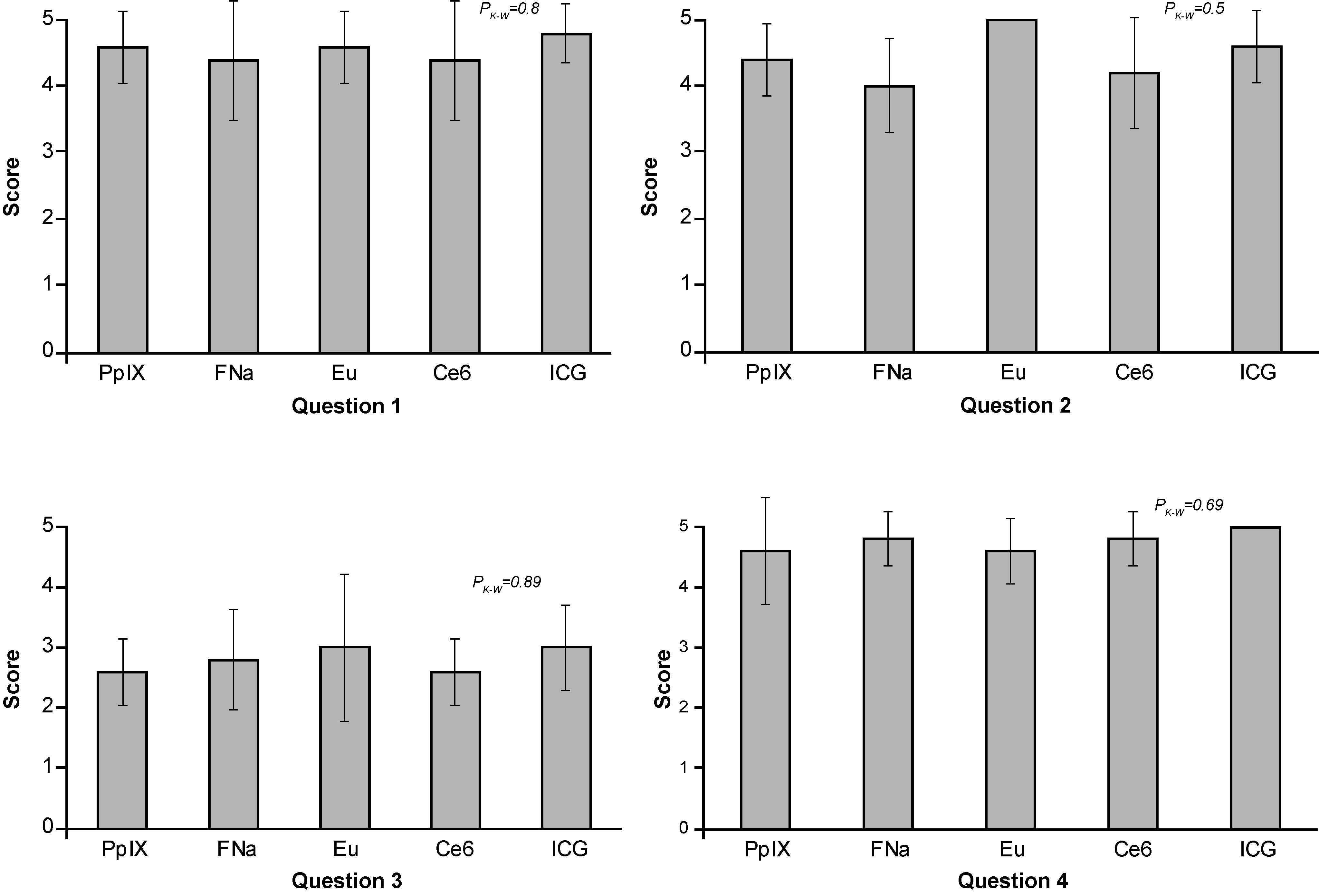

Supplement: Supplemental Figure 3 — Scores for the 4 questions for face validity of the tumor model. Scores for the question “The tumor model replicates real surgery?” ranged from 4.4 to 4.8 (highest score was for ICG); from 4 to 5 for the question “The tumor model is visually similar to real fluorescence-guided tumor surgery?” (highest for europium [Eu]); from 2.6 to 3 (3 meaning the most adequate) for the question “The tumor model is easier/more difficult to perform resection compared to real fluorescence-guided tumor surgery?” (most adequate was ICG); and from 4.6 to 5 for the question “You were successful in completing the task?” (highest for ICG). No comparison reached statistical significance. Used with permission from Barrow Neurological Institute, Phoenix, Arizona. [file Image_3.TIF]

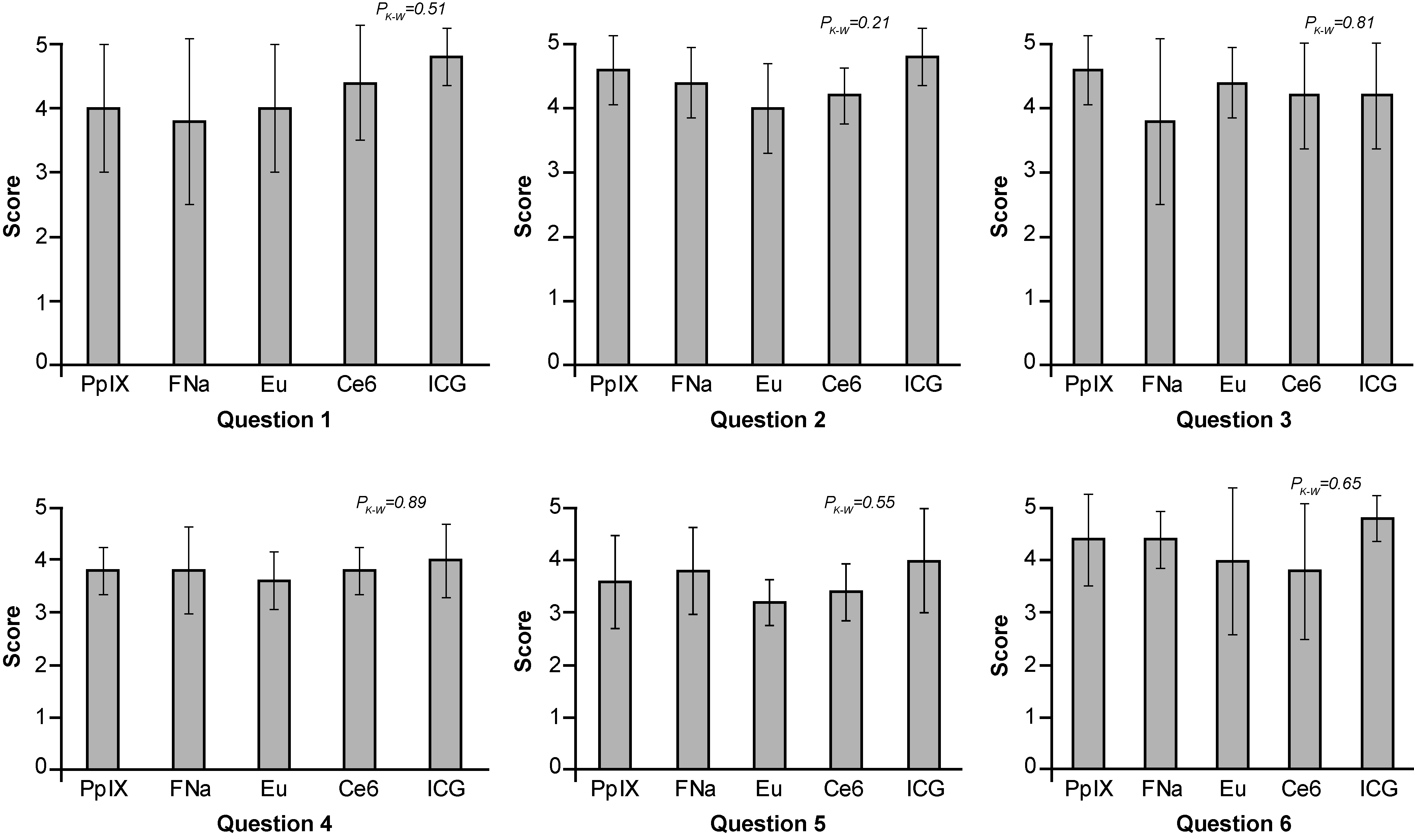

Supplement: Supplemental Figure 4 — Scores for the 6 questions for content validity of the tumor model. Scores for the question “This model improves arachnoidal cortical opening?” ranged from 3.8 to 4.8 (highest score was ICG); from 4 to 4.8 for the question “This model improves debulking with suction?” (highest for ICG); from 3.8 to 4.6 for the question “This model improves subpial resection?” (highest for PpIX); from 3.6 to 4 for the question “This model improves bipolar use?” (highest for ICG); from 3.2 to 4 for the question “This model improves vessel avoidance/management?” (highest for ICG); and from 3.8 to 4.8 for the question “This model allows transference of improved surgical technique when applied to patients?” (highest for ICG). No comparison reached statistical significance. Used with permission from Barrow Neurological Institute, Phoenix, Arizona. [file Image_4.TIF]

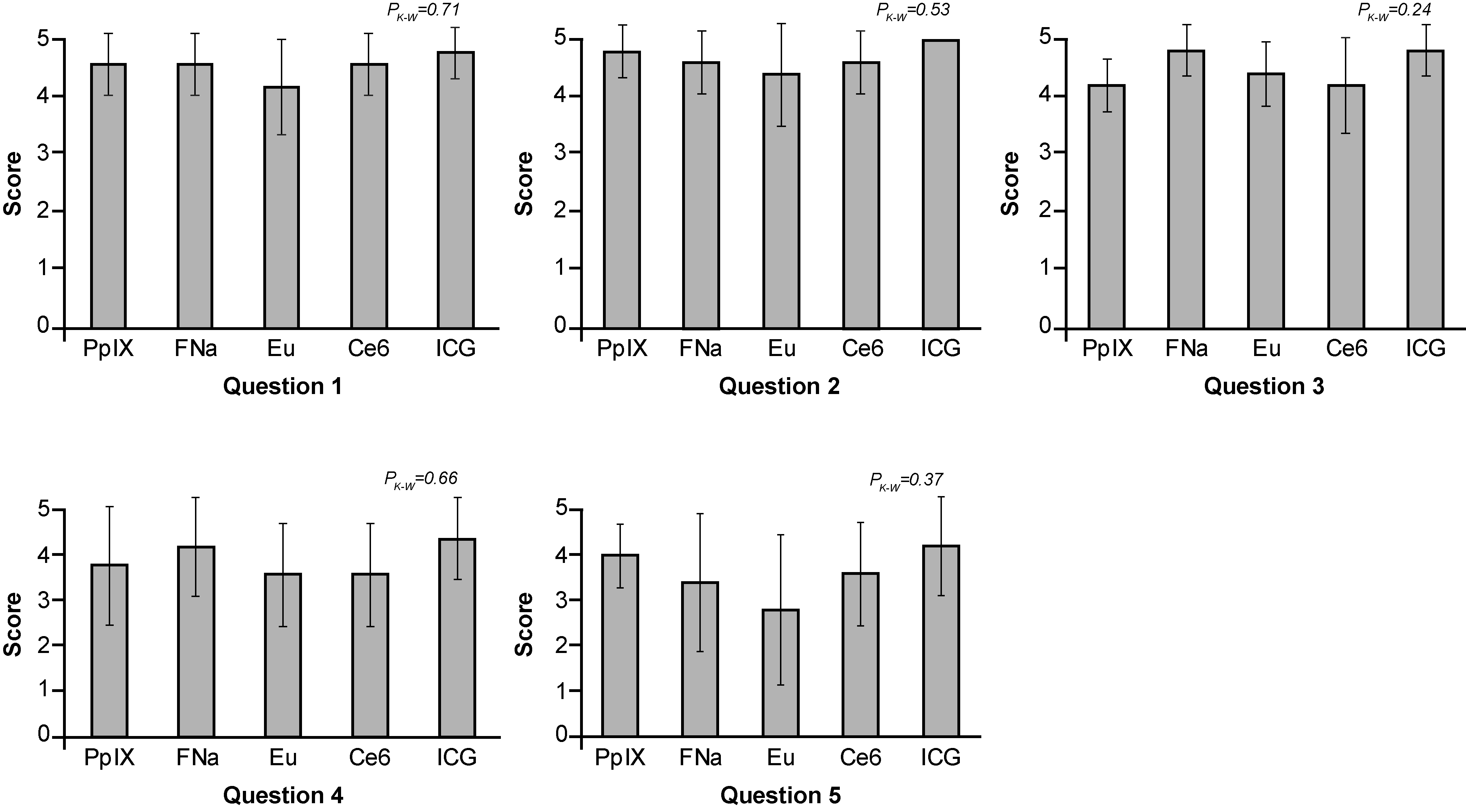

Supplement: Supplemental Figure 5 — Scores for the 5 questions for comparing the fluorophores. The highest and lowest scores for the question “The fluorescence makes resection more convenient for you compared to white light illumination?” were 4.8 (ICG) and 4.2 (europium [Eu]); 5 (ICG) and 4.4 (europium) for the question “The fluorescence makes resection overall more effective compared to white light illumination?”; 4.8 (fluorescein sodium [FNa] and ICG) and 4.2 (chlorin e6 and PpIX) for the question “You find the color of the fluorescence appealing?”; and 4.4 (ICG) and 3.6 (europium and chlorin e6) for the question “You can work in the fluorescence mode continuously without need to switch to white light?” For the question asking participants to rank the fluorophores in order of preference (5 being the best and 1 the worst), ICG had the highest score, followed by PpIX, Ce6, fluorescein sodium, and europium. No comparison reached statistical significance. Used with permission from Barrow Neurological Institute, Phoenix, Arizona. [file Image_5.TIF]
